# Self-Multimerization of mRNA LNP-Derived Antigen Improves Antibody Responses

**DOI:** 10.3390/vaccines14010080

**Published:** 2026-01-12

**Authors:** Cody A. Despins, James Round, Lisa Dreolini, Tracy S. Lee, Scott D. Brown, Robert A. Holt

**Affiliations:** 1 Department of Basic and Translational Research, BC Cancer Research Institute, 675 W 10th Ave, Vancouver, BC V5Z 1L3, Canada; 2 Department of Molecular Biology and Biochemistry, Simon Fraser University, SSB8166—8888 University Drive, Burnaby, BC V5A 1S6, Canada; 3 Interdisciplinary Oncology Program, University of British Columbia, 675 W 10th Ave, Vancouver, BC V5Z 1L3, Canada; 4 Department of Medical Genetics, University of British Columbia, C201—4500 Oak Street, Vancouver, BC V6H 3N1, Canada

**Keywords:** antigen multimerization, vaccination, mRNA LNP, vaccine development

## Abstract

**Background**: mRNA LNP technology is now being widely applied as a highly effective vaccine platform. Antigen multimerization is a well-established approach to enhance the antibody titers and protective efficacy of several protein subunit vaccines. However, this approach has been less explored for mRNA LNP vaccines. **Methods**: Here, within the context of mRNA LNP vaccination, we used mStrawberry (mSb) as a model antigen to conduct a comprehensive, head-to-head comparison of the ability of the foldon (3-mer), IMX313 (7-mer), and ferritin (24-mer) multimerization domains to enhance immunogenicity in mice. **Results**: We compared multimerized antigen to monomeric secreted antigen and monomeric surface-displayed antigen and observed that the IMX313 domain efficiently multimerized mSb protein and significantly enhanced anti-mSb antibody titers, whereas the foldon and ferritin domains failed to multimerize or improve antibody levels. **Conclusions**: Our results extend the observation of improved immunogenicity from antigen multimerization to mRNA LNP vaccines and indicate that the 7-mer forming IMX313 multimerization domain may be an ideal candidate for multimer formation in the context of mRNA LNP vaccination. Future studies are needed to evaluate the multimerization of pathogen-derived antigens, in the mRNA LNP format, for the enhancement of neutralization and protective efficacy.

## 1. Introduction

Widespread use of mRNA LNP vaccines during the COVID-19 pandemic (mRNA-1273 and BNT162b2) demonstrated the mRNA LNP platform’s capacity for both rapid development (critical for pandemic response and updating for variant emergence) and high protective efficacy. Since its debut in this setting, mRNA LNP vaccine development has expanded to target many other pathogens. mRESVIA (mRNA-1345) demonstrated an 83.7% protection from RSV-associated lower respiratory tract disease (LRTD), with ≥2 signs/symptoms in older adults in a phase II/III clinical trial [1], and was approved for use in older adults (≥60 years of age) by the FDA in 2024, representing the first non-SARS-CoV-2 mRNA LNP vaccine approval. In 2025, mRESVIA was further approved by the FDA for adults (18–59 years of age) with increased risk for LRTD, and it is currently being evaluated for use in children, another population with high risk of severe outcomes from RSV, in clinical trials [2,3]. A multiantigen CMV mRNA LNP vaccine (mRNA-1647) is currently being evaluated in a phase III clinical trial following promising phase I and II results [4,5]. A seasonal influenza mRNA LNP vaccine demonstrated superior immunogenicity variables to conventional protein vaccine comparators in phase III clinical trials and is also being evaluated as a flu/COVID-19 combination vaccine (mRNA-1010 and mRNA-1083, respectively) [6]. Many mRNA LNP vaccines targeting other viruses, including those for VSV, HIV, Zika, mpox, rabies, and EBV, are in the earlier stages of clinical trial evaluation [7]. Further, mRNA LNP vaccines for bacterial targets, such as *Borrelia* spp. (the causative agent of lyme disease), and parasites, such as *Plasmodium falciparum* (the causative agent of malaria), are also in clinical trials [7]. These examples highlight the extensive innovation in infectious disease vaccine development enabled by mRNA LNP technology.

In addition to infectious disease applications, the ability to rapidly develop and test mRNA LNP vaccines makes personalized neoantigen vaccines a more viable therapeutic option in the field of oncology. Once a patient’s tumor is sequenced and putative neoantigens are identified, neoantigen-encoding mRNA LNP vaccines can be formulated and delivered to boost anti-tumor immunity, often in combination with other immunotherapies. This strategy has recently shown promising results in clinical trials for pancreatic cancer [8] and melanoma [9]. Furthermore, several non-personalized cancer mRNA LNP vaccines encoding common cancer-associated antigens are also being developed [7].

Overall, mRNA LNP technology has revolutionized modern vaccinology and promises major reduction in disease burden in both infectious disease and oncology as well as potential for further improvement and applications as the technology develops.

All vaccines rely on being sufficiently immunogenic to drive the expansion of target-specific populations to a degree such that there is reduction or prevention of disease upon subsequent exposure (for infectious disease applications) or to promote re-engagement of immunity with the tumor (for oncology applications). Thus, in both settings, generating sufficient immunogenicity is vital and, towards this goal, it is well established that vaccination using a multimerized antigen can enhance antibody responses. There are two primary proposed mechanisms for this: firstly, increased antigen avidity through multimerization can enhance B cell activation [10], and secondly, a soluble antigen of an appropriate size (10–100 nm) can transit to draining lymph nodes independent of antigen-presenting cells (APCs) [11,12,13], thereby increasing the concentration of antigen and likelihood of interactions with reactive B cells.

Several domains are commonly used to achieve antigen multimerization. The foldon domain, derived from fibritin of bacteriophage T4 [14], mediates the formation of a 3-mer and has demonstrated enhanced neutralization of pseudoviruses compared to non-foldon vaccines and complete protection against lethal (10 × LD_50_) challenge in murine models for influenza [15]. Foldon has also been applied for SARS-CoV [16], MERS-CoV [17], SARS-CoV-2 [18], RSV [19], and JEV vaccines [20]. Similarly, the IMX313 domain, which is derived from the chicken C4bp oligomerization domain [21] and mediates the formation of a 7-mer, has been shown to significantly increase IgG levels at low doses and improve parasite transmission blockage compared to monomeric protein in a Pfs25-targeted malaria vaccine [22]. Lastly, the ferritin multimerization domain, commonly derived from *H. pylori*, mediates the formation of a 24-mer. An RBD-encoding ferritin vaccine approach increased antigen uptake by macrophages and DCs and increased antibody titers and proportions of reactive CD8^+^ and CD4^+^ T cells following vaccination, compared to monomeric RBD [23]. Ferritin has also been used in vaccines for influenza [24,25], EBV [26,27], HIV [28], Zika [29], and Lyme disease [30].

Multimerization using foldon, IMX313, ferritin, and other domains has been well characterized and broadly applied for protein vaccines. Similar findings have been reported for pDNA vaccines [31,32] and viral vector vaccines [22]. However, the evaluation of antigen multimerization in the context of mRNA LNP vaccines remains limited. Whether the mRNA LNP platform is fully amendable to antigen multimerization (i.e., whether the dynamics of mRNA LNP-derived expression allow antigens to self-multimerize at a meaningful rate within the cell), if there are important considerations compared to multimerization for protein vaccines and other vaccine types, and whether some multimerization domains may be better suited for mRNA LNP application are important questions to address towards the development of more effective mRNA LNP vaccines.

In this study, we aimed to address this knowledge gap by evaluating multimerization in the setting of mRNA LNP vaccination. Using mSb as a representative antigen of interest, we tested three commonly used multimerization domains (foldon [3-mer], IMX313 [7-mer], and ferritin [24-mer]) and evaluated their capacity to multimerize, secrete from the cell, and enhance antibody responses induced by mRNA LNP vaccination.

## 2. Materials and Methods

### 2.1. Multimer Structural Predictions

Alphafold 2.3.1 [33] was used to predict structures for multimerized mSb constructs with foldon (3-mer), IMX313 (7-mer), and ferritin (24-mer). Each prediction run used one NVIDIA RTX A6000 GPU, 32 CPUs, and 220 GB RAM. The “-m multimer” parameter was used. For foldon and IMX313, protein reference files were provided with 3 and 7 copies of the construct sequence, respectively. For ferritin, to make the prediction computationally tractable, 24 copies of the ferritin sequence were predicted in a multimer run and, separately, 1 copy of the mSb-Ferritin construct was predicted. The highest confidence model for each prediction run was selected. For mSb-Ferritin, the alpha-carbons of the ferritin portion of the monomer were aligned to the structure of each of the multimer 24-mer ferritins using ChimeraX (version 1.5), generating the final multimerized structure.

### 2.2. Plasmid DNA Generation

DNA sequences for all plasmids used in this study are listed in Supplementary Data File S1. Coding sequences were codon-optimized using GenSmart (accessed 26 September 2022) (GenScript [Piscataway, NJ, USA]) and then manually edited to remove uridine residues in the wobble position of two-family codons. RNA structures were predicted by RNAFold [34] and coding sequences were modified as needed to maintain an optimal RNA structure in the 5′ UTR. All golden gate assembly parts were domesticated to remove Type IIs restriction sites and then incorporated into pNL and/or pLZ1/4. To create the pRNA series of plasmids, pRNA-Destination plasmids were combined with appropriate pLZ plasmids and assembled in a *PaqC*I-based golden gate assembly reaction performed using the manufacturer’s protocols. In brief, reactions contained 50 ng of destination vector and equimolar amounts of pLZ, 10 U *PaqC*I, 400 U of T4 DNA ligase, and 5 pmoles of *PaqC*I activator in T4 DNA ligase buffer and were cycled 30 times for 37 °C for 5 min, then 16 °C for 5 min. All pDNA constructs were sequence-verified. All enzymes were purchased from New England Biolabs (Ipswich, MA, USA). All golden gate assembly parts were ordered from GenScript, Integrated DNA Technologies (Coralville, IA, USA), or Twist Bioscience (San Francisco, CA, USA).

NEB stable *E. coli* (New England Biolabs, Cat. #C3040H) were used for propagation of DNA. *E. coli* was transformed following manufacturer protocols. *E. coli* was grown in LB broth at 30 °C, shaking at 250 rpm. For solid medium, LB broth was supplemented with Bacto agar (1.5% [*w*/*v*]) (Becton Dickinson [Franklin Lakes, NJ, USA], Cat. #214010) and further supplemented with 100 μg/mL carbenicillin or 50 μg/mL kanamycin, as appropriate. For blue/white screening, media was further supplemented with 1 mM IPTG and 0.1 mg/mL of X-Gal (prepared as a 20 mg/mL stock solution in DMF). All pDNA was quantified by Qubit assay and stored at −20 °C.

### 2.3. mRNA Generation via In Vitro Transcription (IVT)

pDNA template was linearized in preparation for IVT by restriction digest using BspQI (New England Biolabs, Cat. #R0712L) in 1× NEBuffer 3.1 (New England Biolabs, Cat. #B7203) with incubation at 50 °C for ≥1 h. Linearized pDNA was then column-cleaned using the Monarch PCR & DNA cleanup kit (New England Biolabs, Cat. #T1030) following the manufacturer’s protocols and eluting with 50 °C elution buffer. IVT was performed by combining 1000 ng of pDNA with RNA pol reaction buffer (New England Biolabs, Cat. #B9012) (1× final), magnesium acetate (Sigma-Aldrich, [St. Louis, MI, USA], Cat. #63052-100ML) (12.5 mM final), Triton X-100 (Sigma-Aldrich, Cat. #93443) (0.002% final), ATP (New England Biolabs, Cat. #N0450S) (5 mM final), GTP (New England Biolabs, Cat. #N0450S) (5 mM final), CTP (New England Biolabs, Cat. #N0450S) (5 mM final), N1-methyl-pseudouridine-TP (TriLink Biotechnologies [San Diego, CA, USA], Cat. #N-1081-1) (5 mM final), CleanCap Reagent AG (TriLink Biotechnologies, Cat. #N-7113-5) (4 mM final), RNase inhibitor (New England Biolabs, Cat. #M0314L) (10 U/μL final), DTT (Thermo Fisher Scientific [Waltham, MA, USA], Cat. #707265ML) (5 mM final), pyrophosphatase (New England Biolabs, Cat. #M0361L) (0.0025 U/μL final), and T7 polymerase (New England Biolabs, Cat. #M0251) (5 U/μL final) in a total reaction volume of 20 μL (brought to 20 μL with UltraPure dH_2_O [Invitrogen (Waltham, MA, USA), Cat. #10977023]) and incubating at 37 °C for 4–6 h. After incubation, 28 μL of UltraPure dH_2_O and 2 μL of DNAse I (New England Biolabs, Cat. #M0303S) was added; then, samples were incubated at 37 °C for 15 min. Resulting mRNA was then column-cleaned using the Monarch RNA cleanup kit (New England Biolabs, Cat. #T2040) following manufacturer’s protocols with 2 additional wash steps and 5 min of RT incubation before centrifugation steps after the addition of washing or elution buffers. After cleanup, mRNA was quantified using a Qubit BR RNA assay kit (Thermo Fisher Scientific, Cat. #Q10211) and integrity was verified by gel electrophoresis. mRNA was stored at −80 °C.

### 2.4. mRNA LNP Encapsulation and Quantification

For encapsulation, mRNA was diluted to 1 mg/mL with UltraPure dH_2_O (Invitrogen, Cat. #10977023) in a total volume of 22 μL. mRNA was then encapsulated using the NanoAssemblr Spark Hepato9 siRNA kit (Cytiva [Marlborough, MA, USA], Cat. #NWS0009) following manufacturer’s protocols and using 16 μL of Spark Nanoparticle mix. Resulting mRNA LNP was evaluated for concentration and encapsulation efficiency using the Quant-IT RiboGreen RNA assay kit (Thermo Fisher Scientific, Cat. #R11490) based on manufacturer’s protocols with or without the addition of Triton-X 100 (Sigma-Aldrich, Cat. #93443) (2% final) and measured on a VICTOR2 plate reader. All mRNA LNPs were determined to have encapsulation efficiencies of ≥93.6%. Concentration of encapsulated mRNA was used for all further assays. mRNA LNP of each set were generated within 1 day of each other and stored at 4 °C for ≤7 days before use.

### 2.5. Cell Culture

HEK293T/17 cells were cultured in cDMEM (complete DMEM; DMEM [Thermo Fisher Scientific, Cat. #11995073] supplemented with 10% heat-inactivated FBS (Thermo Fisher Scientific, Cat. #A3840302 or Cat. #12484028), 1X GlutaMAX [Thermo Fisher Scientific, Cat. #35050061], 1X Penicillin-Streptomycin [Thermo Fisher Scientific, Cat. #15140122], 1X MycoZap [Lonza (Basel, Switzerland), Cat. #VZA-2031]). HEK293T/17 cells were dissociated using TrypLE Express (Thermo Fisher Scientific, Cat. #12604013) for routine subculturing. K562 cells were cultured in cRPMI (complete RPMI; RPMI 1640 [Thermo Fisher Scientific, Cat. #11875119] supplemented with 10% heat-inactivated FBS, 1X GlutaMAX, 1X Penicillin-Streptomycin, 1X MycoZap, 10 mM HEPES [Thermo Fisher Scientific, Cat. #15630080], 1 mM sodium pyruvate [Thermo Fisher Scientific, Cat. #11360070], and 55 μM 2-mercaptoethanol [Thermo Fisher Scientific, Cat. #21985023]). All cells were grown in an incubator at 37 °C, 5% CO_2_. Cells were cryopreserved in 90% FBS/10% DMSO in vapor-phase liquid nitrogen. HEK293T/17 and K562 cells were purchased from ATCC.

### 2.6. Cell Transfection with mRNA LNP and pDNA

For mRNA LNP transfections of K562 cells, cells were seeded at a density of 600,000 cells/mL in 6-well tissue culture-treated plates (2.5 mL/well) in cRPMI supplemented with 1 μg/mL ApoE4 (Peprotech [Cranbury, NJ, USA], Cat. #350-04). mRNA LNP were adjusted to 8.33 ng/μL with D-PBS (MgCl^−^, CaCl^−^) (Thermo Fisher Scientific, Cat. #14190250) and then cells were transfected by addition of 1.25 μg of mRNA LNP per well dropwise; then, plates were swirled and rocked to disperse complexes. For mock-transfected negative controls, D-PBS (MgCl^−^, CaCl^−^) was used in place of mRNA LNP. For conditions with protein transport inhibition, protein transport inhibitor cocktail (Invitrogen, Cat. #00-4980-83) was diluted in media and added to transfectants at ~1× final concentration at 16 h following transfection.

For pDNA transfections, HEK293T/17 cells were seeded at a density of 140,000 cells/cm^2^ in 6-well tissue culture-treated plates (540,000 cells/mL, 2.5 mL/well) (Corning [Corning, Somerville, MA, USA], Cat. #353046). Transfections were performed at ~16.5 h following seeding. Based on the manufacturer’s recommendations, pDNA (volume normalized with buffer EB [Qiagen (Venlo, The Netherlands), Cat. #19086]) was combined with TransIT-LT reagent (Mirus Bio [Madison, WI, USA], Cat. #MIR2305) at a ratio of 3 μL of TransIT-LT per 1 μg of pDNA per 108 μL of total volume in OptiMEM (Thermo Fisher Scientific, Cat. #3195062) and incubated at RT for ~20 min to allow complex formation. For mock-transfected negative controls, buffer EB alone was used in place of buffer EB/pDNA. Following complex formation, 2.5 μg of pDNA was added dropwise per well, and plates were swirled and/or rocked to disperse complexes.

### 2.7. Flow Cytometry

For assessment of mSb fluorescence alone in transfectants, at ~24 h following transfection, cells were transferred to microfuge tubes, centrifuged at 300× *g* (4 °C), supernatant was removed, and cells were resuspended in D-PBS (MgCl^−^, CaCl^−^) (Thermo Fisher Scientific, Cat. #14190250) and counted on an EVE automated cell counter. A volume equivalent to 1,000,000–1,500,000 cells was then centrifuged at 300× *g* (4 °C), supernatant was removed, and cells were resuspended in 500 μL FACS buffer (5% FBS in D-PBS). Cells were centrifuged at 300× *g* (4 °C), supernatant was removed, and then cells were resuspended in 400 μL of FACS buffer with 0.1 μg/mL DAPI (Sigma-Aldrich, Cat. #D9542).

For assessment of mSb fluorescence and surface mSb expression in transfectants, at ~24 h following transfection, cells were dissociated using 1 mL/plate of enzyme-free cell dissociation buffer (Thermo Fisher Scientific, Cat. #13151014) (to preserve surface proteins). Cells were transferred to microfuge tubes, centrifuged at 300× *g* (4 °C), supernatant was removed, and cells were resuspended in D-PBS (MgCl^−^, CaCl^−^) (Thermo Fisher Scientific, Cat. #14190250) and counted on an EVE automated cell counter. A volume equivalent to ~500,000 cells was then centrifuged at 300× *g* (4 °C), supernatant was removed, and then cells were stained with 100 μL of 1/1000 anti-mSb monoclonal mouse IgG antibody (OriGene [Rockville, MD, USA], Cat. #TA180039) (diluted 1/1000 in FACS buffer [5% FBS in D-PBS]) for 30 min at 4 °C. Cells were then washed in 500 μL FACS buffer twice. Cells were then centrifuged at 300× *g* (4 °C), supernatant was removed, and cells were then stained with 100 μL of 1/200 diluted AlexaFluor647-conjugated goat anti-mouse IgG polyclonal antibody (Biolegend [San Diego, CA, USA], Cat. #405322) (diluted 1/200 in FACS buffer) for 30 min at 4 °C. Cells were then washed in 500 μL FACS buffer twice. Cells were then centrifuged at 300× *g* (4 °C), supernatant was removed, and cells were resuspended in 400 μL of FACS buffer with 0.1 μg/mL DAPI (Sigma-Aldrich, Cat. #D9542).

Following sample preparation, cells were strained through a 0.35 μm FACS tube cap (Corning, Cat. #352235), placed on ice, and analyzed on a Becton Dickinson LSRFortessa flow cytometer.

### 2.8. Western Blot

For assessment of transfectant media in Western blot, at ~24 h following transfection, media from K562 cells was collected from wells by transferring to a microfuge tube. Samples were centrifuged at 300× *g* for 5 min to pellet cells and supernatant was transferred to a new tube twice. cOmplete EDTA-free protease inhibitor (Roche [Basel, Switzerland], Cat. 11873580001) was then added to 1× final. Media samples were then stored at 4 °C for multimer experiments or −20 °C for all other experiments until Western blot analysis.

For assessment of transfectant cell lysate in Western blot, cells were collected at ~24 h following transfection. For HEK293T/17 cells, cells were dissociated using enzyme-free cell dissociation buffer (Thermo Fisher Scientific, Cat. #13151014) (to preserve surface proteins). Cells were then transferred to microfuge tubes, centrifuged at 300× *g*, supernatant was removed, and cells were resuspended in D-PBS (MgCl^−^, CaCl^−^) (Thermo Fisher Scientific, Cat. #14190250) and counted on an EVE automated cell counter. For K562 cells, cells were transferred to microfuge tubes, centrifuged at 300× *g*, supernatant was removed, and cells were resuspended in D-PBS (MgCl^−^, CaCl^−^) (Thermo Fisher Scientific, Cat. #14190250) and counted on an EVE automated cell counter. A volume equivalent to ~500,000 cells was then centrifuged at 300× *g*, supernatant was removed, and cells pellets were stored at −20 °C. Cell pellets were then thawed and resuspended in 100 μL of RIPA buffer (Thermo Fisher Scientific, Cat. #89900) with cOmplete EDTA-free protease inhibitor (Roche, Cat. #11873580001) added to 1–1.25× final. Samples were then incubated on ice for 15 min to lyse, then centrifuged at 14,000× *g* for 15 min at 4 °C. Supernatant was then transferred to a new tube. Lysed cell pellets and prepared cell lysates were stored at 20 °C. As needed, lysed cell pellets were re-centrifuged and supernatant was re-transferred to generate more prepared cell lysate sample.

Transfectant media or prepared cell lysate samples were then combined with a 1:3 volume ratio with Laemmli buffer (4×) (BioRad [Hercules, CA, USA], Cat. #1610747) supplemented with (reducing) or without (non-reducing) 2-mercaptoethanol (1.42 M final) (BioRad, Cat. #1610710), or with (reducing) DTT (50 mM final). Samples were heated (85 °C for sample sets with multimers, 95 °C for 5 min for sets without multimers) and then 5 μL were loaded per lane onto a 4–15% mini-PROTEAN TGX polyacrylamide gel (BioRad, Cat. #4561085 or 4561086) and ran at 50 V for 15–30 min; then, 150 V for 35–60 min in 1× Tris/Glycine/SDS running buffer (BioRad, Cat. #1610732). Proteins were then transferred to nitrocellulose blots in transfer buffer (1× Tris/Glycine [BioRad, Cat. #1610734], with 20% methanol) at 0.30 A for 1.33–1.5 h on ice. After transfer, blots were cut as needed, washed 1× in 10 mL TBS-T (TBS with 0.1% Tween-20), then blocked with 10 mL of EveryBlot blocking buffer (BioRad, Cat. #12010020) for 10 min at RT. Blots were then washed 2× in 10 mL TBS-T and then probed with 10 mL of mouse anti-StrepTagII monoclonal antibody (diluted to 1/5000) (LSBio, Cat. #LS-C413454), mouse anti-mSb monoclonal antibody (diluted 1/2000) (OriGene, Cat. #TA180039), or rabbit anti-vinculin monoclonal antibody (diluted 1/5000) (Abcam [Cambridge, UK], Cat. #ab129002) (all dilutions were in EveryBlot blocking buffer) overnight at 4 °C. Blots were then washed 4 times in 10 mL TBS-T and probed with 10 mL of HRP-conjugated goat anti-mouse IgG polyclonal antibody (diluted 1/10,000 in EveryBlot blocking buffer) (Jackson Immunology [West Grove, PA, USA], Cat. #115035003) or HRP-conjugated goat anti-rabbit IgG polyclonal antibody (diluted 1/10,000 in EveryBlot blocking buffer) (Jackson Immunology, Cat. #111035003), as appropriate, for 1 h at RT. Blots were then washed 4 times in 10 mL TBS-T, developed using Clarity Western ECL substrate (BioRad, Cat. #1705060) and then imaged on a BioRad ChemiDoc imaging system. SeeBluePlus2 pre-stained protein standard (Invitrogen, Cat. #LC5925) or HiMark pre-stained protein standard (Invitrogen, Cat. #LC5699) was run alongside samples to evaluate molecular weight.

### 2.9. Mice

Female C57BL/6J-A0201 (C57BL/6J-Mcph1^Tg(HLA-A2.1)1Enge^/J [Jackson strain #003475]) mice were purchased from Jackson Laboratory (Bar Harbor, ME, USA) and allowed to acclimate for ≥7 days following arrival. Mice were 7–8 weeks of age at the beginning of experiments. Each group consisted of 4 mice. All experiments involving animals were performed within the BC Cancer Animal Resource Centre (ARC) under specific pathogen-free conditions. 

### 2.10. mRNA LNP Vaccinations

mRNA LNP vaccines were adjusted to 50 ng/μL using D-PBS (MgCl^−^, CaCl^−^) (Thermo Fisher Scientific, Cat. #14190250). Immediately prior to injection, mRNA LNP vaccines were warmed to RT. All vaccinations were performed as a prime-boost regimen, with each mouse receiving 20 μL of mRNA LNP vaccine (1 μg/mouse, intramuscularly) for prime and boost doses. Boost vaccinations were performed 21 days following prime vaccinations and experiment endpoint was 14 days following boost vaccinations.

### 2.11. Serum Isolation

For endpoint plasma/serum, immediately following euthanasia at experimental endpoint by isoflurane followed by CO_2_ inhalation, whole blood was collected via cardiac puncture with a 25G needle. Whole blood was placed in an SST microtainer tube (Becton Dickinson, Cat. #365967), clotted for 30–60 min at RT, and then placed on ice/at 4 °C. Clotted whole blood was then centrifuged at 2000× *g* for 10 min at 4 °C and the supernatant was then transferred to a new tube and stored at −80 °C.

### 2.12. Enzyme-Linked Immunosorbent Assay (ELISA)

Immulon 2HB plates (Thermo Fisher Scientific, Cat. #14-245-61) were coated by addition of 100 μL/well of 2 μg/mL of recombinant mStrawberry (OriGene, Cat. #TP790044) in ELISA carbonate coating buffer (Invitrogen, Cat #CB01100) and incubation overnight at 4 °C. Plates were washed 3 times with 200 μL/well of 1× wash buffer (Invitrogen, Cat. #WB01, then blocked by addition of 200 μL/well of 1× ELISA buffer (Invitrogen, Cat. #DS98200) and incubation at RT for 3 h. Plates were then washed 3 times with 200 μL/well of 1× wash buffer. Next, 100 μL/well of individual serum samples from vaccinated mice or negative control C57BL/6J-A0201 serum (Jackson Laboratory, custom product) serially diluted in 1× ELISA buffer (Invitrogen, Cat. #DS98200) were added and plates were incubated overnight at 4 °C. Plates were then washed 3 times with 200 μL/well of 1× wash buffer and 100 μL/well of 0.2 μg/mL HRP-conjugated donkey anti-mouse IgG polyclonal antibody (Invitrogen, Cat. #A16017) diluted in 1× ELISA buffer was then added and plates were incubated for 2 h at RT. Plates were then washed 3 times with 200 μL/well of 1× wash buffer and 100 μL/well of TMB stabilized chromogen (Thermo Fisher Scientific, Cat. #SB01) was then added. Reactions were allowed to develop for ~3 min and were then stopped by addition of 100 μL/well of ELISA stop solution (Invitrogen, Cat. #SS03). OD_450_ values were read on a Molecular Devices SpectraMax iD3 microplate reader, then adjusted to account for path length (dividing by the correction factor of 0.596, corresponding to a volume of 200 μL/well). All buffers were diluted to 1× using UltraPure dH_2_O (Invitrogen, Cat. #10977023). ANOVA analysis was performed in R (v4.5.2) in RStudio (v2025.05.2, Build 517). Raw ELISA data is provided in Supplementary Data File S2.

## 3. Results

### 3.1. Construct Design and Antigen Structure Prediction

Plasmid DNA (pDNA) constructs for generating mRNA for encapsulation and transfection/vaccination were designed incorporating several features for enhanced antigen expression, immunogenicity, and ease of use. These constructs have a T7 promoter followed by an AG initiator for use with TriLink CleanCap AG during in vitro transcription (IVT) reactions to generate 5′ Cap-1 (m7GpppN1mp) capped mRNA (which, as opposed to Cap-0 [m7GpppN1p], has an additional 2′-O-methylation at the nucleotide 1 position). Such Cap-1 capping has been shown to be essential in mimicking self mRNA of higher eukaryotes, which has 2′-O-methylation at the nucleotide 1 position and thereby reduces type I interferon activation and translational inhibition typically induced by uncapped or Cap-0 mRNAs [35,36,37]. The construct is also designed such that the IVT transcript has a 5′ α-globin UTR and a eukaryotic Kozak sequence motif for efficient protein translation [38,39] and 2 × 3′ β-globin untranslated regions (UTRs) for greater mRNA translation/stability [40]. mRNA CDS were codon-optimized using GenSmart codon optimization to enhance translation efficiency. The poly-A tail is 120 bp to promote translation/stability [40], bisected to reduce pDNA recombination [41] and directly incorporated into the plasmid sequence (rather than added following IVT via enzymatic polyadenylation, which results in inconsistent polyA lengths [40]). The construct was also designed for linearization prior to IVT via a type IIS restriction enzyme (SapI or BspQI), eliminating any 3′ overhang at the end of the polyA tail following linearization, which have been reported to inhibit mRNA translation/stability [40]. Lastly, within the pDNA construct, an upstream CMV promotor enables expression of the mRNA CDS and thereby allows for in vitro assessment of designs by pDNA transfection in mammalian cells, such as HEK293T/17 cells. In addition, the generation of mRNA was also performed using methodology for enhanced antigen expression and immunogenicity—during in vitro transcription, N^1^-methylpseudouridine was incorporated to reduce TLR activation and associated decreases in cellular translation [42].

**Figure 1:**
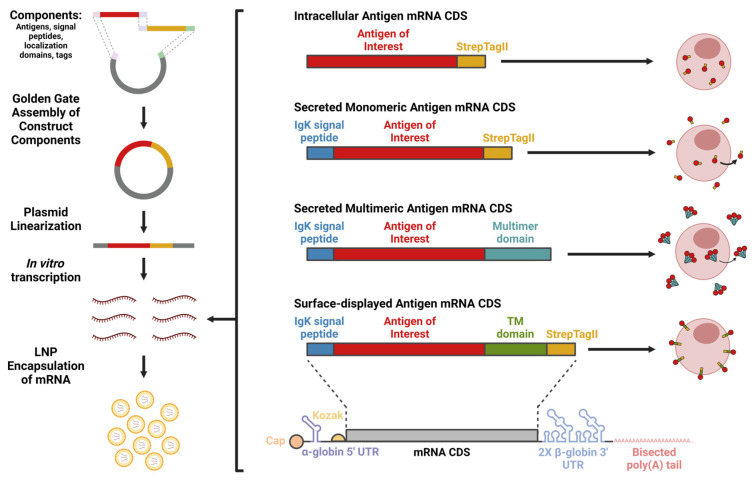
Overview of mRNA LNP vaccine production pipeline and design types. Construct design components such as antigen, signal peptides, domains for localization (such as transmembrane [TM] domains for surface-display), and tags are assembled into a pDNA construct using Golden Gate cloning methods. Plasmids are then isolated, linearized, and in vitro transcription is performed to generate messenger RNA (mRNA). mRNA is encapsulated in lipid nanoparticles (LNP) and evaluated in vitro via transfection and in vivo via intramuscular (IM) vaccination. Four primary mRNA CDS designs were used for: intracellular expression of antigen, expression and secretion of the antigen as a monomer, expression and secretion of the antigen as a multimer (3-mer foldon-based multimer shown), and expression and surface-display of the antigen. The figure was made using BioRender.com.

For the mRNA CDS sequence, four different designs were created: for intracellular antigen expression, for expression and secretion of antigen as a monomer, for expression and secretion of antigen as a multimer, and for expression and surface-display of antigen (Figure 1). Given that mRNA LNP-encoded antigens are expressed in the cytoplasm, the mRNA CDS design for intracellular expression contains the antigen of interest with no additional domains. To stimulate a more robust antibody response, the vaccine-encoded antigen should be accessible to the extracellular environment. Therefore, a murine-derived Igκ signal peptide was added to the N-terminus of the antigen of interest for the mRNA CDS for secretion of monomeric antigen, which targets the encoded protein to the ER for subsequent secretion in mammalian cells [43,44,45]. Multimerization domains (foldon-based, IMX313-based, or ferritin-based) were added in the C-terminal position in combination with Igκ signal peptide to generate an mRNA CDS for secretion of multimeric antigen. Surface-display is a commonly used alternative approach to extracellularly expose antigen. Furthermore, surface-displayed RBD-encoding saRNA LNP vaccines have shown greater antibody titers compared to secreted monomeric RBD [46]. Therefore, designs for surface-display were also created for direct comparison to secreted multimeric antigen designs. For the mRNA CDS for surface-displayed designs, the Igκ signal peptide is included in combination with a C-terminal transmembrane domain, either B7 (including the cytoplasmic tail) or PDGFR-based. In combination with the ER-directing signal peptide Igκ, both the B7 TM domain and PDGFR TM domains have been shown to lead to surface-display of the protein [47,48,49]. Lastly, a StrepTagII sequence was included at the C-terminal end of constructs. This enables the immobilization of antigen for various downstream applications, such as ELISA (using streptavidin-coated plates) or purification. StrepTagII sequences were chosen as tags, rather than other commonly used tags, such as HA or Myc sequences, which are microbe-derived and therefore potentially highly immunogenic. The StrepTagII sequences were not included in the mRNA CDS designs for secretion of multimeric antigen, given concerns that it may interfere with multimerization. For simplicity, these various mRNA CDS are named according to their features from N-terminus to C-terminus (e.g., Igκ-mSb-TM(B7)-Strep) and are referred to as “intracellular”, “secreted monomeric”, “secreted multimeric”, or “surface-displayed” designs.

**Figure 2:**
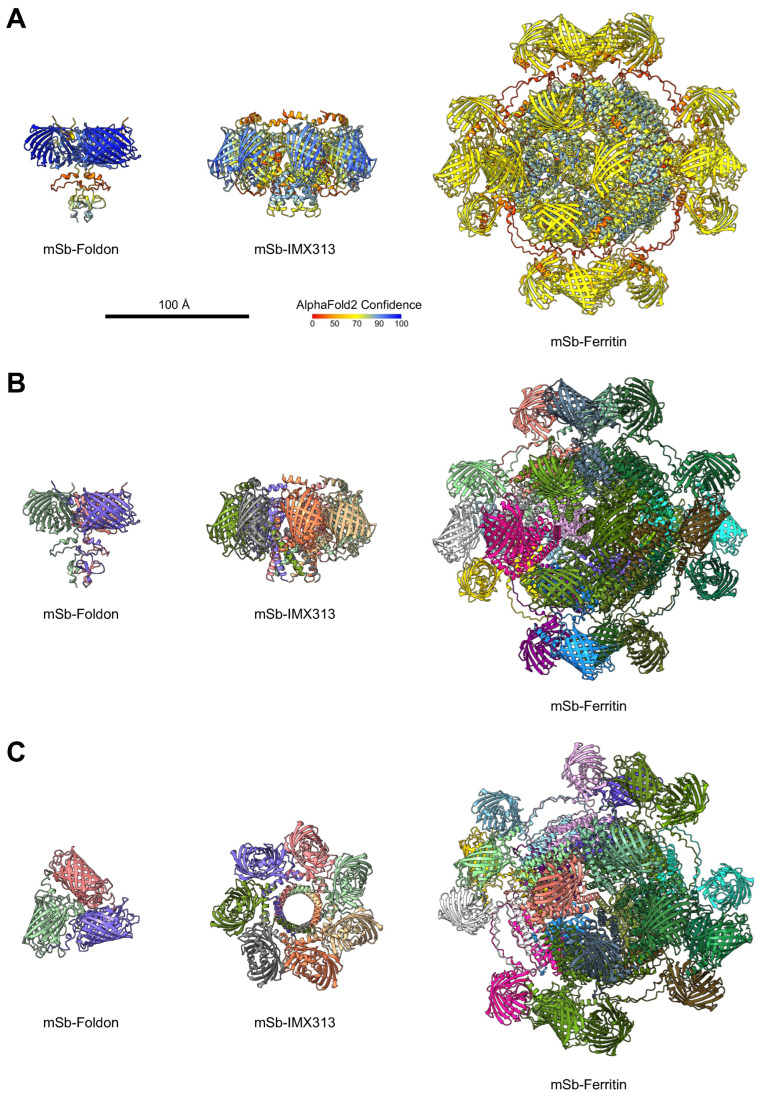
Structural prediction of multimeric mSb designs. mSb multimers were predicted using AlphaFold2 after C-terminal addition of multimerization domains and removal of the IgK signal peptide (mSb-Foldon, left; mSb-IMX313, middle; mSb-Ferritin, right). (**A**) AlphaFold2 confidence of the predicted structures (side-view). (**B**,**C**) Structures with each monomer individually colored (side-view, B; top-view, C). Scale bar applies to all structures.

We firstly performed structural predictions of the different secreted multimeric design types using AlphaFold2 [33] to determine whether the structures were amendable to expected multimer formation (Figure 2). When inspecting the structures of a single subunit of each multimerization domain-containing design alone, each design appeared to have the multimerization domain portion accessible, suggesting multimerization should be possible. Predictions using the multimer parameter supported the ability of the structures to form multimers of 3-mers, 7-mers, and 24-mers for mSb-Foldon, mSb-IMX313, and mSb-Ferritin, respectively, as expected. Additionally, these structures also show the varying geometry of each multimerization domain: foldon generates a “bouquet”-like geometry, IMX313 generates a circular ring geometry, and ferritin generates a spherical geometry. Finally, these data suggest approximate protein particle sizes of 9.5 nm W/L × 6.6 nm H for mSb-Foldon, 11.5 nm W/L × 7.5 nm H for mSb-IMX313, and 22 nm W/L and H for mSb-Ferritin. Therefore, these predicted multimer sizes support APC-independent lymph node drainage (10–100 nm [11,12,13]) as a potential mechanism towards increasing immunogenicity for IMX313 and ferritin multimers.

### 3.2. In Vitro Evaluation of Antigen Designs

**Figure 3:**
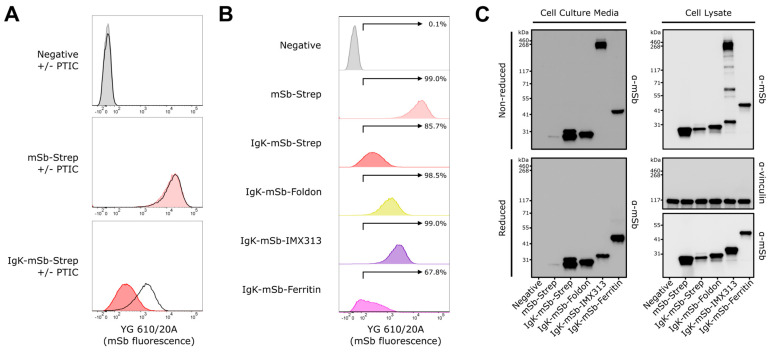
In vitro validation of secreted monomeric and secreted multimeric mSb-encoding mRNA LNP vaccines. (**A**) Flow cytometry analysis of K562 cells transfected with mRNA LNP with and without the addition of protein transport inhibitor cocktail (PTIC). K562 cells were transfected with 1.25 μg of mRNA LNP. At 16 h following transfection, PTIC was added to respective conditions (black line histograms). At 24 h following transfection (i.e., 8 h following addition of PTIC), cells were collected and evaluated for mSb fluorescence by flow cytometry. Events were gated to isolate live singlet cells. Data is representative of two replicates (one replicate shown). See Supplemental Figure S1 for gating strategy. (**B**) Flow cytometry analysis following transfection of K562 cells with mRNA LNP. K562 cells were transfected with 1.25 μg of mRNA LNP. At 24 h following transfection, cells were collected and evaluated for mSb fluorescence by flow cytometry. Events were gated to isolate live singlet cells. Data is representative of two replicates (one replicate shown). See Supplemental Figure S1 for gating strategy. (**C**) Western blot analysis of K562 cells transfected with 1.25 μg mRNA LNP. At 24 h following transfection, cells (for preparing cell lysate) and culture media were collected. Samples were run on SDS-PAGE in reducing or non-reducing conditions, transferred to nitrocellulose, probed via anti-mSb and anti-vinculin (loading control) antibodies. Blots were then probed with appropriate HRP-conjugated secondary antibodies, then developed and imaged.

Secreted monomeric and secreted multimeric designs were then evaluated in vitro for expression/secretion and expression/secretion/multimerization, respectively (Figure 3). For these experiments, mSb-Strep (intracellular) served as a negative control for evaluating secretion. K562 cells were transfected with mSb-Strep (intracellular), Igκ-mSb-Strep (secreted monomeric) mRNA LNP, or mock-transfected (negative control for transfection/mSb expression) with or without addition of protein transport inhibitor cocktail (PTIC); then, mSb fluorescence was evaluated by flow cytometry (Figure 3A). PTIC contains brefeldin A and monensin which are well-established inhibitors of the export pathway in mammalian cells [50,51]. The addition of PTIC had no effect on mock-transfected negative control cells or mSb-Strep mRNA LNP-transfected cells. Conversely, the addition of PTIC in Igκ-mSb-Strep mRNA LNP-transfected cells resulted in increased mSb fluorescence. Given that inhibition of secretion led to intracellular accumulation of mSb, these data indicate the inclusion of the N-terminal Igκ signal peptide successfully leads to secretion in our designs. All mRNA LNP successfully led to mSb expression—transfection resulted in mSb fluorescent positivity of 99.0% for mSb-Strep, 85.7% for Igκ-mSb-Strep, 98.5% for Igκ-mSb-Foldon, 99.0% for Igκ-mSb-IMX313, and 67.8% for Igκ-mSb-Ferritin, compared to 0.1% positivity in mock-transfected negative controls (Figure 3B).

mSb expression, secretion, and multimerization were evaluated by Western blot analysis of cell lysate and cell culture media samples (Figure 3C). Cell lysate samples run under reducing conditions showed the expected banding of all mSb designs (Igκ = 2.4 kDa, mSb = 26.6 kDa, StrepTagII = 1.1 kDa, foldon = 3.1 kDa, IMX313 = 6.2 kDa, ferritin = 20.0 kDa). Cell culture media samples run under reducing conditions similarly demonstrated expected banding of all mSb designs. Of note, cell culture media of Igκ-mSb-Strep appeared as two similarly sized bands, possibly reflecting a species with post-translational modifications associated with the secretion pathway. Cell culture media of mSb-Strep had only a faintly present band, likely representing release of protein from cells which died/lysed in the period between transfection and collection. Thus, these data further support that the N-terminal Igκ signal peptide successfully leads to antigen secretion, as observed by flow cytometry, and demonstrates successful secretion of Igκ-mSb-Foldon, Igκ-mSb-IMX313, and Igκ-mSb-Ferritin. Given that foldon, IMX313, and ferritin-based multimerization results in 3-mer, 7-mer, and 24-mer products, respectively, a corresponding 3×, 7×, and 24× shift in molecular weight is expected. Comparing reducing and non-reducing conditions, a shift in band size was observed for Igκ-mSb-IMX313, corresponding to the formation of a 7-mer product (32 kDa × 7 = 224 kDa). Additionally, the presence of only the 7-mer band in the cell media sample of Igκ-mSb-IMX313 suggests complete multimerization of these monomers prior to secretion and that the multimer is relatively stable in the extracellular environment. The presence of multiple other bands in the cell lysate sample of Igκ-mSb-IMX313 possibly reflects a stepwise nature of multimer formation. No bands corresponding to multimers were observed for Igκ-mSb-Foldon or Igκ-mSb-Ferritin. Under non-reducing or reducing conditions, multimeric and monomeric forms, respectively, have been observed for foldon-based [52], IMX313-based [22,53], and ferritin-based multimers [52]. Therefore, under conditions permissive to multimer detection, we find no evidence of multimerization for IgK-mSb-Foldon or IgK-mSb-Ferritin.

**Figure 4:**
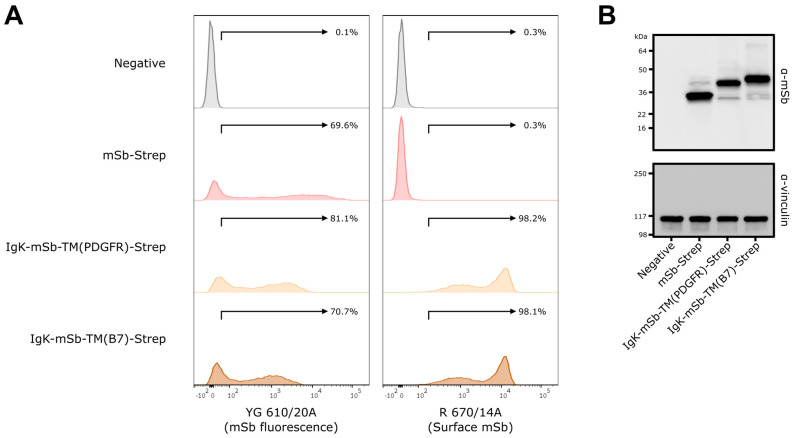
In vitro validation of surface-displayed mSb-encoding pDNA constructs used to generate mRNA LNP vaccines. (**A**) Flow cytometry analysis of HEK293T/17 cells transfected with 2.5 μg of pDNA. At 24 h following transfection, cells were collected and stained with anti-mSb and then AlexaFluor647-conjugated secondary antibodies, then were evaluated for mSb fluorescence and surface staining by flow cytometry. Events were gated to isolate live singlet cells. Data is representative of two replicates (one replicate shown). See Supplemental Figure S2 for gating strategy. (**B**) Western blot analysis of HEK293T/17 cells transfected with 2.5 μg pDNA. At 24 h post-transfection, cells (for preparing cell lysate) were collected. Cell lysates were prepared and run on SDS-PAGE in reducing conditions, transferred to nitrocellulose, and then probed via anti-mSb and anti-vinculin (loading control) antibodies. Blots were then probed with appropriate HRP-conjugated secondary antibodies, then developed and imaged.

Next, surface-displayed designs were evaluated in vitro for expression and surface-display (Figure 4). For these experiments, mSb-Strep (intracellular) served as a negative control for evaluating surface-display. HEK293T/17 cells were transfected with mSb-Strep, Igκ-mSb-TM(PDGFR)-Strep, or Igκ-mSb-TM(B7)-Strep pDNA or mock-transfected (negative control for transfection/mSb expression) and then mSb fluorescence and mSb surface staining was evaluated by flow cytometry (Figure 4A). Transfection resulted in mSb fluorescent positivity of 69.6% for mSb-Strep, 81.1% for Igκ-mSb-TM(PDGFR)-Strep, and 70.7% for Igκ-mSb-TM(B7)-Strep, compared to 0.1% positivity in mock-transfected negative controls, indicating all designs successfully lead to mSb expression. Alternatively, surface staining was detected at a positivity of 0.3% for mSb-Strep, 98.2% for Igκ-mSb-TM(PDGFR)-Strep, and 98.1% for Igκ-mSb-TM(B7)-Strep, compared to 0.3% positivity in mock-transfected negative controls. These data showed that the inclusion of the N-terminal Igκ signal peptide with a PDGFR or B7 TM domain results in successful surface-display.

As a secondary validation of expression, Western blot analysis was performed on cell lysates of mSb-Strep, Igκ-mSb-TM(PDGFR)-Strep, or Igκ-mSb-TM(B7)-Strep transfectants (Figure 4B). A dominant band corresponding to expected sizes for all mSb designs was observed (Igκ = 2.4 kDa, mSb = 26.6 kDa, TM(PDGFR) = 5.4 kDa, TM(B7) = 7.7 kDa, StrepTagII = 1.1 kDa). Igκ-mSb-TM(B7)-Strep was selected for further evaluation given the ~7.6-fold longer half-life of B7-based surface-display compared to PDGFR-based surface-display [54].

### 3.3. In Vivo Evaluation of Antigen Designs

**Figure 5:**
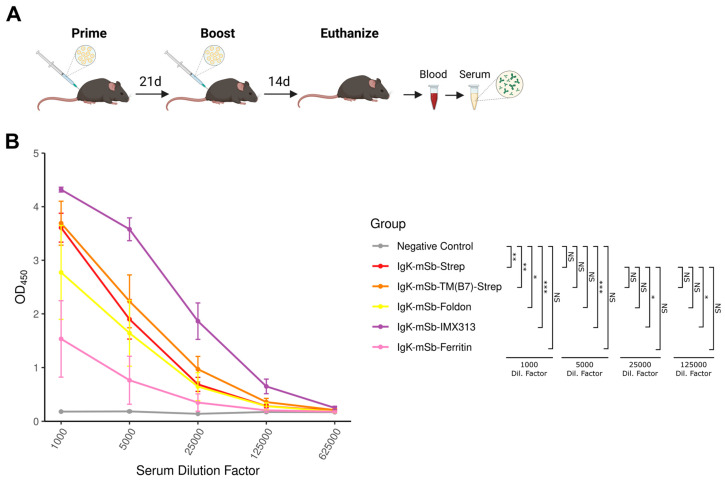
In vivo evaluation of secreted monomeric, secreted multimeric, and surface-displayed mSb-encoding mRNA LNP vaccines. (**A**) In vivo study design for testing of mSb-encoding mRNA LNP vaccination in C57BL/6J-A0201 mice (*n* = 4 mice/group). The figure was made using BioRender.com. (**B**) Indirect ELISA analysis of negative control C57BL/6J-A0201 serum (*n* = 3) and endpoint serum isolated from vaccinated mice of each mRNA LNP vaccine type (*n* = 4 mice/group). Immulon 2HB plates were coated overnight with 100 μL/well of 2 μg/mL recombinant mSb and blocked; then, individual mouse serum was added at various dilutions and incubated overnight and plates were then incubated with HRP-conjugated donkey anti-mouse IgG secondary antibodies, developed using TMB, and the OD450 was read. Points represent the mean and error bars represent SEM. Significance was determined using a one-way ANOVA with Tukey’s HSD (NS, *p* > 0.05; *, *p* ≤ 0.05; **, *p* ≤ 0.01; ***, *p* ≤ 0.001).

Next, to determine the relative immunogenicity of the various mSb-encoding designs, an in vivo prime-boost vaccination study was performed in C57BL/6-A0201 mice, comparing mRNA LNP vaccines of the following designs: Igκ-mSb-Strep, Igκ-mSb-TM(B7)-Strep, Igκ-mSb-Foldon, Igκ-mSb-IMX313, or Igκ-mSb-Ferritin (Figure 5A) (*n* = 4 mice/group). Of note, the mice used were transgenic for HLA-A*02:01^+^ so as to allow future assessment of MHC class I epitopes presented by this allele, which is beyond the scope of the present study. At experimental endpoint (14 days following boost vaccination), blood samples were collected, and serum was isolated. To assess immunogenicity following vaccination, an indirect ELISA using recombinant mSb-coated plates was performed for the serum of each mouse (Figure 5B). All vaccines elicited responses compared to negative control serum samples derived from the same mouse strain (*p* ≤ 0.05; ANOVA), except Igκ-mSb-Ferritin. No significant difference was observed between Igκ-mSb-Strep and Igκ-mSb-TM(B7)-Strep (*p* > 0.05), indicating secretion and surface-display are equally immunogenic in this context. Similarly, Igκ-mSb-Foldon or Igκ-mSb-Ferritin showed no difference (*p* > 0.05) in antigen-specific IgG relative to the Igκ-mSb-Strep. Assuming this lack of increased antigen-specific IgG was due to lack of multimerization, these findings are in agreement with our in vitro observations. Alternatively, Igκ-mSb-IMX313 generated ~5-fold greater antigen-specific IgG compared to its monomeric counterpart (*p* ≤ 0.05). These data indicate that high rates of antigen self-multimerization can be achieved from mRNA LNP, translating to improved antibody responses, and suggest that IMX313 may be well-suited as a multimerization domain in the context of mRNA LNP vaccination.

## 4. Discussion

Compared to protein vaccines, reports of the use of multimerization domains in mRNA LNP vaccines are limited. BNT162b1 was a SARS-CoV-2 mRNA LNP vaccine candidate developed by Pfizer/BioNTech, which encoded the Spike protein receptor binding domain (RBD) with a foldon multimerization domain to generate trimeric multimers for secretion and was evaluated in a phase I/II clinical trial (though ultimately not rolled out due to increased reactogenicity compared to BNT162b2, despite similar level of antibody responses) [55,56]. While this candidate demonstrated multimerization in vitro following mRNA LNP transfection [18], there was no in vivo evaluation compared to a secreted monomeric control that would enable conclusions as to the possible benefits of multimer formation. However, similar secreted trimeric foldon-based RBD designs delivered as mRNA LNP have been directly compared in vivo to monomeric equivalents (secreted monomeric RBD, as well as surface-displayed native spike) and demonstrated superior neutralizing titer and strain cross-reactivity in mice [57]. Similarly, ferritin-based multimerization of trimeric RBD subunits significantly increased neutralization titers and IFN-γ reactive cells compared to trimeric RBD subunits without the ferritin domain in mRNA LNP-vaccinated mice [52]. Alternatively, others report only limited proportions of properly folded multimeric antigen (0.7%) for ferritin-based designs delivered as mRNA, as well as reduced in vitro and in vivo expression and reduced immunogenicity of such designs compared to surface-displayed comparators [58]. In our data, foldon- and ferritin-based designs did not multimerize to a detectable level and did not improve immunogenicity. Thus, the mechanisms allowing foldon and ferritin domains to multimerize and enhance immunogenicity of mRNA LNP vaccines in some contexts and not others require further investigation.

To the best of our knowledge, our data represents the first evaluation of the IMX313 multimerization domain in the context of mRNA LNP vaccination. Of particular note, in this study, the IMX313-containing design, Igκ-mSb-IMX313, was found to multimerize in vitro and increase antibody responses in vivo, while foldon- and ferritin-containing designs (Igκ-mSb-Foldon and Igκ-mSb-Ferritin) did not. These data suggest that, at least for some antigens, the IMX313 multimerization domain may be superior to foldon and ferritin in the setting of mRNA LNP vaccines. We propose three possible explanations for this finding: higher abundance level, greater inter-monomer affinity, and lack of structural interference. Firstly, the IMX313-containing design had greater abundance following transfection than any other secreted design. It is possible this enhanced abundance level directly drove, at least in part, the observed enhanced immunogenicity. Alternatively, effective multimerization may be dependent on a critical threshold of abundance. Increased intracellular antigen concentration would promote the likelihood of interactions between subunits, necessary for multimer formation. It is unclear as to why the IMX313-containing design had increased abundance levels, but this may be related to increased solubility, as has been reported for other protein fusions such as MBP fusions [59]. Further, high abundance levels may be particularly important for secreted antigen, given the limited time between antigen translation and secretion (after which antigen concentration will much lower in the extracellular environment and therefore less likely to multimerize). Alternatively, the IMX313 domain may have greater inter-monomer affinity, and therefore form more stable multimers that remain bound together during and following secretion. Stable multimer formation may also be more important in situations of lower cellular antigen abundance that would reduce the likelihood of reformation following dissociation. Lastly, there may be structural interference for specific combinations of antigen and multimerization domain (i.e., mSb in combination with IMX313 may have allowed uninhibited multimer formation, whereas there may have been occlusion of the foldon and ferritin multimer domain by the mSb portion of the chimeric protein). While we performed structural predictions with the goal of selecting candidate mSb/multimer domain combinations without evidence of such issues, the imperfect nature of these predictions makes this remain a possible consideration.

Future work may expand upon our results. Firstly, we selected mSb as a representative antigen candidate of interest, given its fluorescence makes it readily detected; however, as mentioned above, alternative antigens may be better suited for some multimerization domains, therefore these findings should be evaluated in additional contexts. Similarly, we selected three widely used multimerization domains (foldon, IMX313, and ferritin) with varying geometries, sizes, and orders of multimerization (3-, 7-, 24-mers). Future studies evaluating additional multimerization domains in the context of mRNA LNPs may reveal commonalities between those best-suited for mRNA LNP vaccine application. In addition, increased immunogenicity from multimerization does not necessarily directly correlate with increased protection. The capacity of multimeric designs to target key regions of pathogen-derived antigens and similarly evoke enhanced neutralization and enhanced protection should be explored in future work. Lastly, we used secreted multimeric antigen designs and focused on antibody responses induced by these vaccines, given that antibody titer/neutralizing titer are the primary correlates of protection for vaccines against many infectious diseases [60] and antibody responses are most suitable for our applications of interest. However, a limitation of our study is that vaccine-induced T cell responses were not evaluated, and can be critical for protection in some disease contexts, such as some intracellular pathogens and cancer. Assessment of the impact of multimerization on such responses may be beneficial for certain vaccine applications. In principle, surface-displayed antigen may generate better CD8^+^ T cell responses as the antigen is retained by the cell and can be processed and presented on MHC I. Therefore, developing multimeric forms of surface-displayed antigen may have significant potential, inducing stronger antibody responses through possible synergistic effects of surface-display and multimerization while also being retained for MHC I presentation to induce CD8^+^ T cell responses, and should be explored in future studies.

## 5. Conclusions

In conclusion, our data supports the applicability of multimerization domains in mRNA LNP vaccine design and highlights IMX313 as a potential preferential domain for mRNA LNPs. However, further research using pathogen-derived antigens is needed to identify the factors that lead to successful multimerization, and to demonstrate protective efficacy, before these types of multimerization strategies can be incorporated into mRNA LNP vaccine development efforts.

## Data Availability

The raw data supporting the conclusions of this article will be made available by the authors on request.

## Supplementary Materials

The following supporting information can be downloaded at: https://www.mdpi.com/article/10.3390/vaccines14010080/s1.

## References

[1] Wilson E., Goswami J., Baqui A.H., Doreski P.A., Perez-Marc G., Zaman K., Monroy J., Duncan C.J.A., Ujiie M., Rämet M. (2023). Efficacy and Safety of an MRNA-Based RSV PreF Vaccine in Older Adults. N. Engl. J. Med..

[2] Schnyder Ghamloush S., Qian H., Chen H., Whitten J., Stoszek S.K., Snape M.D. (2025). 168. Safety, Reactogenicity, and Immunogenicity of MRNA-1345, an Investigational Respiratory Syncytial Virus Vaccine, in Participants 2 to <18 Years at High Risk of Severe Disease. Proc. Open Forum Infect. Dis..

[3] Hall C.B., Weinberg G.A., Iwane M.K., Blumkin A.K., Edwards K.M., Staat M.A., Auinger P., Griffin M.R., Poehling K.A., Erdman D. (2009). The Burden of Respiratory Syncytial Virus Infection in Young Children. N. Engl. J. Med..

[4] Fierro C., Brune D., Shaw M., Schwartz H., Knightly C., Lin J., Carfi A., Natenshon A., Kalidindi S., Reuter C. (2024). Safety and Immunogenicity of a Messenger RNA–Based Cytomegalovirus Vaccine in Healthy Adults: Results from a Phase 1 Randomized Clinical Trial. J. Infect. Dis..

[5] Panther L., Basnet S., Fierro C., Brune D., Leggett R., Peterson J., Pickrell P., Lin J., Wu K., Lee H. (2023). 2892. Safety and Immunogenicity of MRNA-1647, an MRNA-Based Cytomegalovirus Vaccine in Healthy Adults: Results of a Phase 2, Randomized, Observer-Blind, Placebo-Controlled, Dose-Finding Trial. Proc. Open Forum Infect. Dis..

[6] Soens M., Ananworanich J., Hicks B., Lucas K.J., Cardona J., Sher L., Livermore G., Schaefers K., Henry C., Choi A. (2025). A Phase 3 Randomized Safety and Immunogenicity Trial of MRNA-1010 Seasonal Influenza Vaccine in Adults. Vaccine.

[7] Żak M.M., Zangi L. (2025). Clinical Development of Therapeutic MRNA Applications. Mol. Ther..

[8] Rojas L.A., Sethna Z., Soares K.C., Olcese C., Pang N., Patterson E., Lihm J., Ceglia N., Guasp P., Chu A. (2023). Personalized RNA Neoantigen Vaccines Stimulate T Cells in Pancreatic Cancer. Nature.

[9] Weber J.S., Carlino M.S., Khattak A., Meniawy T., Ansstas G., Taylor M.H., Kim K.B., McKean M., Long G.V., Sullivan R.J. (2024). Individualised Neoantigen Therapy MRNA-4157 (V940) plus Pembrolizumab versus Pembrolizumab Monotherapy in Resected Melanoma (KEYNOTE-942): A Randomised, Phase 2b Study. Lancet.

[10] Abbott R.K., Crotty S. (2020). Factors in B Cell Competition and Immunodominance. Immunol. Rev..

[11] Schudel A., Francis D.M., Thomas S.N. (2019). Material Design for Lymph Node Drug Delivery. Nat. Rev. Mater..

[12] Roth G.A., Picece V.C.T.M., Ou B.S., Luo W., Pulendran B., Appel E.A. (2022). Designing Spatial and Temporal Control of Vaccine Responses. Nat. Rev. Mater..

[13] Bagby T.R., Cai S., Duan S., Thati S., Aires D.J., Forrest L. (2012). Impact of Molecular Weight on Lymphatic Drainage of a Biopolymer-Based Imaging Agent. Pharmaceutics.

[14] Güthe S., Kapinos L., Möglich A., Meier S., Grzesiek S., Kiefhaber T. (2004). Very Fast Folding and Association of a Trimerization Domain from Bacteriophage T4 Fibritin. J. Mol. Biol..

[15] Du L., Leung V.H.C., Zhang X., Zhou J., Chen M., He W., Zhang H.Y., Chan C.C.S., Poon V.K.M., Zhao G. (2011). A Recombinant Vaccine of H5N1 HA1 Fused with Foldon and Human IgG Fc Induced Complete Cross-Clade Protection against Divergent H5N1 Viruses. PLoS ONE.

[16] Li J., Ulitzky L., Silberstein E., Taylor D.R., Viscidi R. (2013). Immunogenicity and Protection Efficacy of Monomeric and Trimeric Recombinant SARS Coronavirus Spike Protein Subunit Vaccine Candidates. Viral Immunol..

[17] Tai W., Zhao G., Sun S., Guo Y., Wang Y., Tao X., Tseng C.T.K., Li F., Jiang S., Du L. (2016). A Recombinant Receptor-Binding Domain of MERS-CoV in Trimeric Form Protects Human Dipeptidyl Peptidase 4 (HDPP4) Transgenic Mice from MERS-CoV Infection. Virology.

[18] Vogel A.B., Kanevsky I., Che Y., Swanson K.A., Muik A., Vormehr M., Kranz L.M., Walzer K.C., Hein S., Güler A. (2021). BNT162b Vaccines Protect Rhesus Macaques from SARS-CoV-2. Nature.

[19] Rainho-Tomko J.N., Pavot V., Kishko M., Swanson K., Edwards D., Yoon H., Lanza L., Alamares-Sapuay J., Osei-Bonsu R., Mundle S.T. (2022). Immunogenicity and Protective Efficacy of RSV G Central Conserved Domain Vaccine with a Prefusion Nanoparticle. Npj Vaccines.

[20] Han X., Cai Z., Dai Y., Huang H., Cao X., Wang Y., Fang Y., Liu G., Zhang M., Zhang Y. (2022). Re-Burying Artificially Exposed Surface of Viral Subunit Vaccines Through Oligomerization Enhances Vaccine Efficacy. Front. Cell Infect. Microbiol..

[21] Ogun S.A., Dumon-Seignovert L., Marchand J.B., Holder A.A., Hill F. (2008). The Oligomerization Domain of C4-Binding Protein (C4bp) Acts as an Adjuvant, and the Fusion Protein Comprised of the 19-Kilodalton Merozoite Surface Protein 1 Fused with the Murine C4bp Domain Protects Mice against Malaria. Infect. Immun..

[22] Li Y., Leneghan D.B., Miura K., Nikolaeva D., Brian I.J., Dicks M.D.J., Fyfe A.J., Zakutansky S.E., De Cassan S., Long C.A. (2016). Enhancing Immunogenicity and Transmission-Blocking Activity of Malaria Vaccines by Fusing Pfs25 to IMX313 Multimerization Technology. Sci. Rep..

[23] Ma X., Zou F., Yu F., Li R., Yuan Y., Zhang Y., Zhang X., Deng J., Chen T., Song Z. (2020). Nanoparticle Vaccines Based on the Receptor Binding Domain (RBD) and Heptad Repeat (HR) of SARS-CoV-2 Elicit Robust Protective Immune Responses. Immunity.

[24] Kanekiyo M., Wei C.J., Yassine H.M., McTamney P.M., Boyington J.C., Whittle J.R.R., Rao S.S., Kong W.P., Wang L., Nabel G.J. (2013). Self-Assembling Influenza Nanoparticle Vaccines Elicit Broadly Neutralizing H1N1 Antibodies. Nature.

[25] Houser K.V., Chen G.L., Carter C., Crank M.C., Nguyen T.A., Burgos Florez M.C., Berkowitz N.M., Mendoza F., Hendel C.S., Gordon I.J. (2022). Safety and Immunogenicity of a Ferritin Nanoparticle H2 Influenza Vaccine in Healthy Adults: A Phase 1 Trial. Nat. Med..

[26] Kanekiyo M., Bu W., Joyce M.G., Meng G., Whittle J.R.R., Baxa U., Yamamoto T., Narpala S., Todd J.P., Rao S.S. (2015). Rational Design of an Epstein-Barr Virus Vaccine Targeting the Receptor-Binding Site. Cell.

[27] Li P., Jiang Z., Shi J., Sha H., Yu Z., Zhao Y., Han S., Ma L. (2024). A Self-Assembled Nanoparticle Vaccine Elicits Effective Neutralizing Antibody Response against EBV Infection. Front. Immunol..

[28] Sliepen K., Ozorowski G., Burger J.A., Van Montfort T., Stunnenberg M., LaBranche C., Montefiori D.C., Moore J.P., Ward A.B., Sanders R.W. (2015). Presenting Native-like HIV-1 Envelope Trimers on Ferritin Nanoparticles Improves Their Immunogenicity. Retrovirology.

[29] Pattnaik A., Sahoo B.R., Struble L.R., Borgstahl G.E.O., Zhou Y., Franco R., Barletta R.G., Osorio F.A., Petro T.M., Pattnaik A.K. (2023). A Ferritin Nanoparticle-Based Zika Virus Vaccine Candidate Induces Robust Humoral and Cellular Immune Responses and Protects Mice from Lethal Virus Challenge. Vaccines.

[30] Kamp H.D., Swanson K.A., Wei R.R., Dhal P.K., Dharanipragada R., Kern A., Sharma B., Sima R., Hajdusek O., Hu L.T. (2020). Design of a Broadly Reactive Lyme Disease Vaccine. Npj Vaccines.

[31] Lainšček D., Fink T., Forstnerič V., Hafner-Bratkovič I., Orehek S., Strmšek Ž., Manček-Keber M., Pečan P., Esih H., Malenšek Š. (2021). A Nanoscaffolded Spike-RBD Vaccine Provides Protection against SARS-CoV-2 with Minimal Anti-Scaffold Response. Vaccines.

[32] Lin H., Jiang Y., Li Y., Zhong Y., Chen M., Jiang W., Xiang R., Cao N., Sun L., Wang X. (2025). Ferritin-Based HA DNA Vaccine Outperforms Conventional Designs in Inducing Protective Immunity Against Seasonal Influenza. Vaccines.

[33] Jumper J., Evans R., Pritzel A., Green T., Figurnov M., Ronneberger O., Tunyasuvunakool K., Bates R., Žídek A., Potapenko A. (2021). Highly Accurate Protein Structure Prediction with AlphaFold. Nature.

[34] Gruber A.R., Lorenz R., Bernhart S.H., Neuböck R., Hofacker I.L. (2008). The Vienna RNA Websuite. Nucleic Acids Res..

[35] Daffis S., Szretter K.J., Schriewer J., Li J., Youn S., Errett J., Lin T.Y., Schneller S., Zust R., Dong H. (2010). 2′-O Methylation of the Viral MRNA Cap Evades Host Restriction by IFIT Family Members. Nature.

[36] Hornung V., Ellegast J., Kim S., Brzózka K., Jung A., Kato H., Poeck H., Akira S., Conzelmann K.-K., Schlee M. (2006). 5’-Triphosphate RNA Is the Ligand for RIG-I. Science.

[37] Kumar P., Sweeney T.R., Skabkin M.A., Skabkina O.V., Hellen C.U.T., Pestova T.V. (2014). Inhibition of Translation by IFIT Family Members Is Determined by Their Ability to Interact Selectively with the 5’-Terminal Regions of Cap0-, Cap1- and 5’ppp- MRNAs. Nucleic Acids Res..

[38] Abendure J.B., Babendure J.L., Ing J.-H., Tsien R.Y. (2006). Control of Mammalian Translation by MRNA Structure near Caps. RNA.

[39] Kozak M. (1987). An Analysis of S’-Noncoding Sequences from 699 Vertebrate Messenger RNAs. Nucleic Acids Res..

[40] Holtkamp S., Kreiter S., Selmi A., Simon P., Koslowski M., Huber C., Türeci Ö., Sahin U. (2006). Modification of Antigen-Encoding RNA Increases Stability, Translational Efficacy, and T-Cell Stimulatory Capacity of Dendritic Cells. Blood.

[41] Trepotec Z., Geiger J., Plank C., Aneja M.K., Rudolph C. (2019). Segmented Poly(A) Tails Significantly Reduce Recombination of Plasmid DNA without Affecting MRNA Translation Efficiency or Half-Life. RNA.

[42] Andries O., Mc Cafferty S., De Smedt S.C., Weiss R., Sanders N.N., Kitada T. (2015). N1-Methylpseudouridine-Incorporated MRNA Outperforms Pseudouridine-Incorporated MRNA by Providing Enhanced Protein Expression and Reduced Immunogenicity in Mammalian Cell Lines and Mice. J. Control. Release.

[43] Cheng K.-W., Wang F., Lopez G.A., Singamsetty S., Wood J., Dickson P.I., Chou T.-F. (2021). Evaluation of Artificial Signal Peptides for Secretion of Two Lysosomal Enzymes in CHO Cells. Biochem. J..

[44] Wang X., Liu H., Yuan W., Cheng Y., Han W. (2016). Efficient Production of CYTL1 Protein Using Mouse IgGκ Signal Peptide in the CHO Cell Expression System. Acta Biochim. Biophys. Sin..

[45] Güler-Gane G., Kidd S., Sridharan S., Vaughan T.J., Wilkinson T.C.I., Tigue N.J. (2016). Overcoming the Refractory Expression of Secreted Recombinant Proteins in Mammalian Cells through Modification of the Signal Peptide and Adjacent Amino Acids. PLoS ONE.

[46] Komori M., Nogimori T., Morey A.L., Sekida T., Ishimoto K., Hassett M.R., Masuta Y., Ode H., Tamura T., Suzuki R. (2023). SaRNA Vaccine Expressing Membrane-Anchored RBD Elicits Broad and Durable Immunity against SARS-CoV-2 Variants of Concern. Nat. Commun..

[47] Lin Y.-C., Lu C.B.-M., Su W.-C., Prijovich C.-I.M. (2013). The B7-1 Cytoplasmic Tail Enhances Intracellular Transport and Mammalian Cell Surface Display of Chimeric Proteins in the Absence of a Linear ER Export Motif. PLoS ONE.

[48] Liao K.-W., Lo Y.-C., Roffler S.R. (2000). Activation of Lymphocytes by Anti-CD3 Single-Chain Antibody Dimers Expressed on the Plasma Membrane of Tumor Cells. Gene Ther..

[49] Liao K.W., Chen B.M., Liu T.B., Tzou S.C., Lin Y.M., Lin K.F., Su C.I., Roffler S.R. (2003). Stable Expression of Chimeric Anti-CD3 Receptors on Mammalian Cells for Stimulation of Antitumor Immunity. Cancer Gene Ther..

[50] Klausner R.D., Donaldson J.G., Lippincott-Schwartz J. (1992). Brefeldin A: Insights into the Control of Membrane Traffic and Organelle Structure. J. Cell Biol..

[51] Mollenhauer H.H., Morré D.J., Rowe L.D. (1990). Alteration of Intracellular Traffic by Monensin; Mechanism, Specificity and Relationship to Toxicity. Biochim. Et Biophys. Acta.

[52] Sun W., He L., Zhang H., Tian X., Bai Z., Sun L., Yang L., Jia X., Bi Y., Luo T. (2021). The Self-Assembled Nanoparticle-Based Trimeric RBD MRNA Vaccine Elicits Robust and Durable Protective Immunity against SARS-CoV-2 in Mice. Signal Transduct. Target. Ther..

[53] Malhi H., Homad L.J., Wan Y.H., Poudel B., Fiala B., Borst A.J., Wang J.Y., Walkey C., Price J., Wall A. (2022). Immunization with a Self-Assembling Nanoparticle Vaccine Displaying EBV GH/GL Protects Humanized Mice against Lethal Viral Challenge. Cell Rep. Med..

[54] Liao K.-W., Chou W.-C., Lo Y.-C., Roffler S.R. (2001). Design of Transgenes for Efficient Expression of Active Chimeric Proteins on Mammalian Cells. Biotechnol. Bioeng..

[55] Walsh E.E., Frenck R.W., Falsey A.R., Kitchin N., Absalon J., Gurtman A., Lockhart S., Neuzil K., Mulligan M.J., Bailey R. (2020). Safety and Immunogenicity of Two RNA-Based Covid-19 Vaccine Candidates. N. Engl. J. Med..

[56] Mulligan M.J., Lyke K.E., Kitchin N., Absalon J., Gurtman A., Lockhart S., Neuzil K., Raabe V., Bailey R., Swanson K.A. (2020). Phase I/II Study of COVID-19 RNA Vaccine BNT162b1 in Adults. Nature.

[57] Liang Q., Wang Y., Zhang S., Sun J., Sun W., Li J., Liu Y., Li M., Cheng L., Jiang Y. (2022). RBD Trimer MRNA Vaccine Elicits Broad and Protective Immune Responses against SARS-CoV-2 Variants. iScience.

[58] Mu Z., Whitley J., Martik D., Sutherland L., Newman A., Barr M., Parks R., Wiehe K., Cain D.W., Hodges K.Z. (2024). Comparison of the Immunogenicity of MRNA-Encoded and Protein HIV-1 Env–Ferritin Nanoparticle Designs. J. Virol..

[59] Kapust R.B., Waugh D.S. (1999). Escherichia Coli Maltose-binding Protein Is Uncommonly Effective at Promoting the Solubility of Polypeptides to Which It Is Fused. Protein Sci..

[60] Plotkin S.A. (2010). Correlates of Protection Induced by Vaccination. Clin. Vaccine Immunol..

