# Supplementary figures and images for: Self-Multimerization of mRNA LNP-Derived Antigen Improves Antibody Responses
Source: Vaccines (Basel). 2026 Jan 12;14(1):80. doi: 10.3390/vaccines14010080 (PMC12846620; doi:10.3390/vaccines14010080)

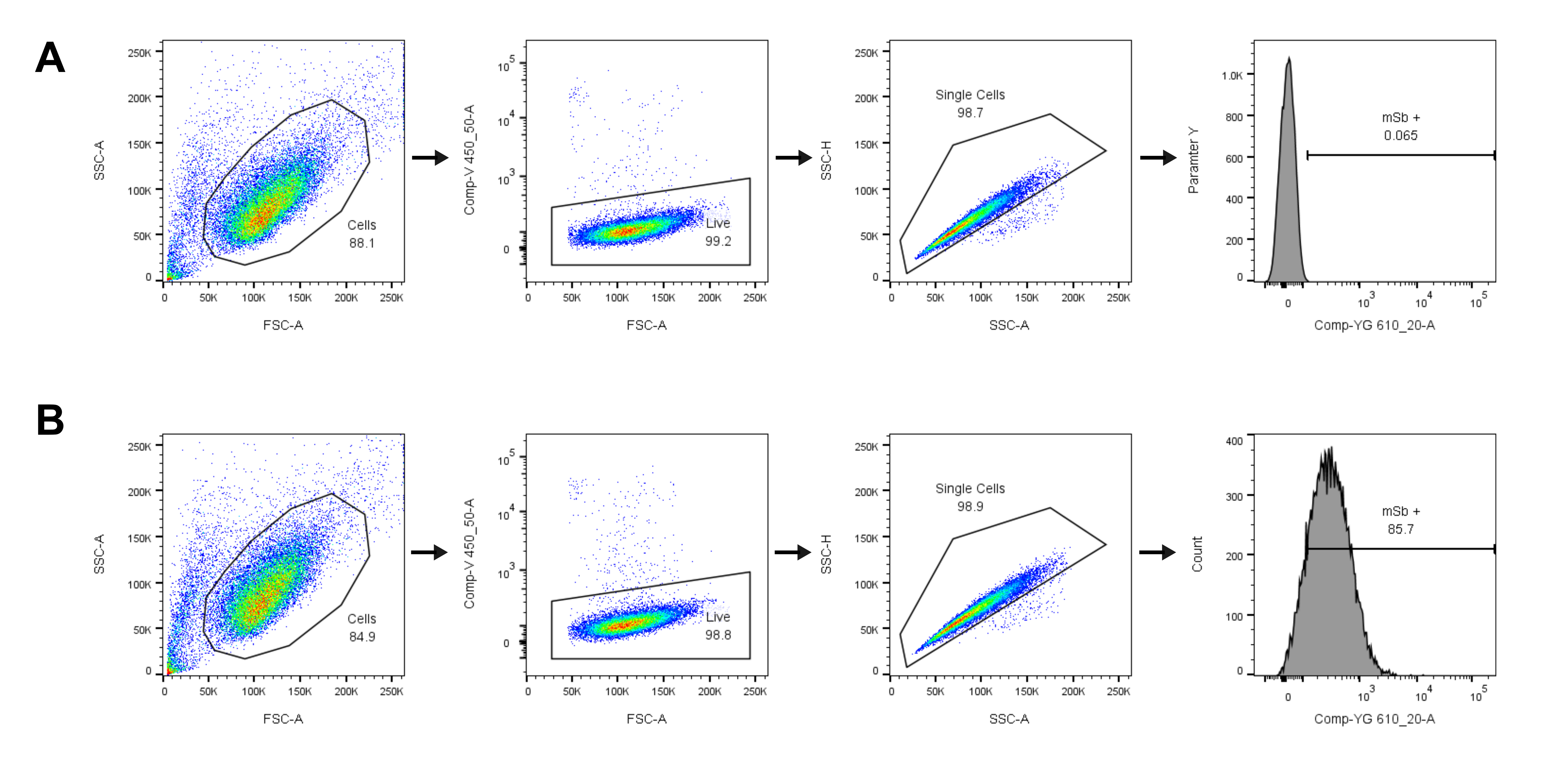

Supplement: Supplementary file 1 [file vaccines-14-00080-s001.zip › FigureS1.png]

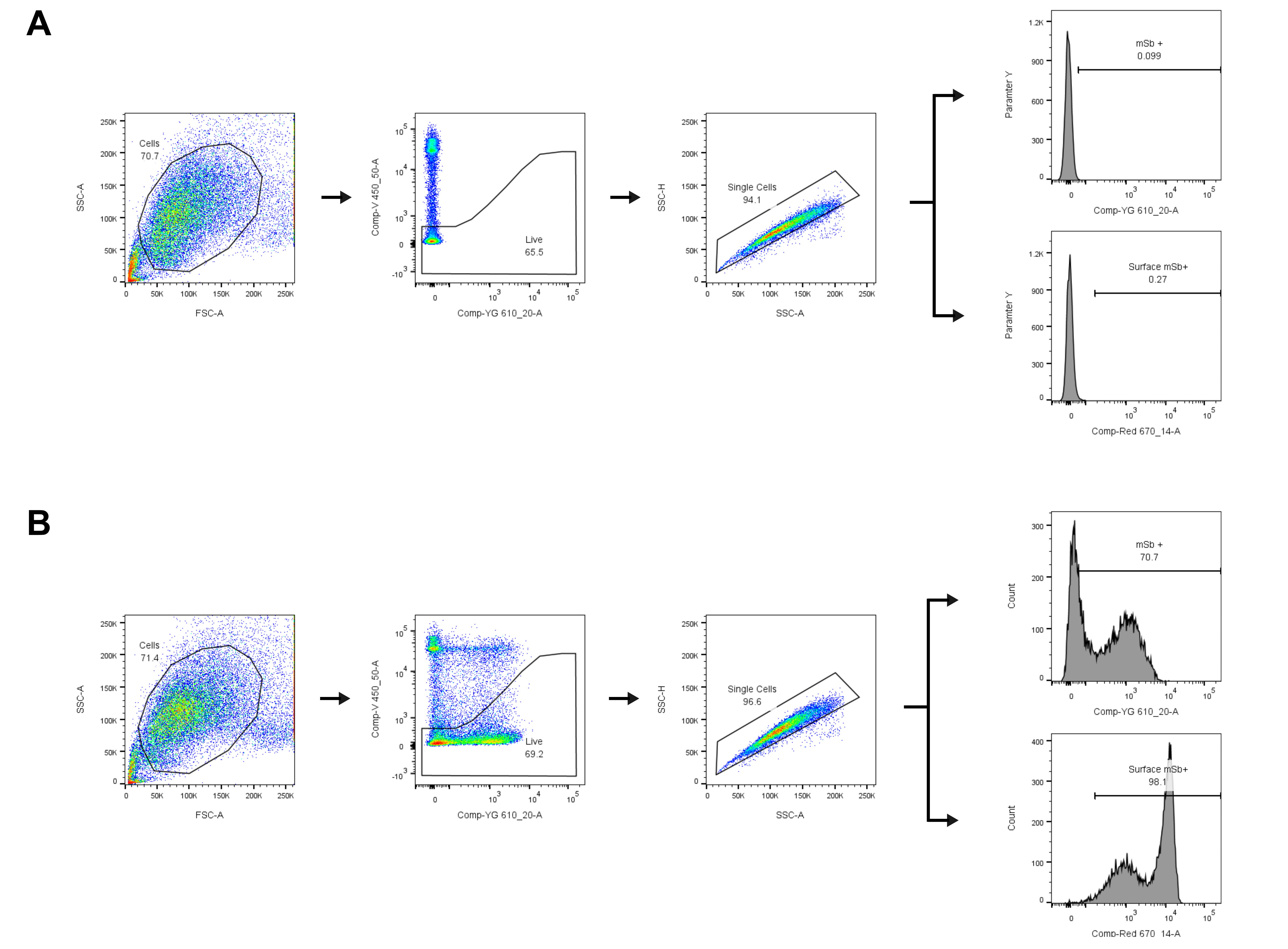

Supplement: Supplementary file 1 [file vaccines-14-00080-s001.zip › FigureS2.png]
